# The impact of treatment with eluxadoline on health-related quality of life among adult patients with irritable bowel syndrome with diarrhea

**DOI:** 10.1007/s11136-018-2008-z

**Published:** 2018-09-28

**Authors:** Jessica L. Abel, Robyn T. Carson, David A. Andrae

**Affiliations:** Allergan plc, 5 Giralda Farms, Madison, NJ 07940 USA

**Keywords:** Irritable bowel syndrome, Diarrhea, Health-related quality of life, Eluxadoline, IBS-QOL

## Abstract

**Purpose:**

Irritable bowel syndrome with diarrhea (IBS-D) significantly impacts health-related quality of life (HRQOL). This post hoc analysis of two phase III trials evaluated the effects of eluxadoline treatment on disease-specific HRQOL among patients with IBS-D.

**Methods:**

Adult patients meeting Rome III criteria for IBS-D were randomized to oral eluxadoline (75 mg or 100 mg) or placebo twice daily in two phase III clinical trials for 52 weeks (IBS-3001) and 26 weeks (IBS-3002). The Irritable Bowel Syndrome Quality of Life (IBS-QOL) questionnaire assessed disease-specific HRQOL throughout the study. Changes from baseline to Week 26 in IBS-QOL total and subscale scores were analyzed using an analysis of covariance model. Percentages of IBS-QOL responders with ≥ 14- and 20-point changes were evaluated for IBS-QOL total and subscale scores. A longitudinal mixed-effects model was fitted to evaluate mean IBS-QOL total scores. A cumulative distribution function for change from baseline to Week 26 in IBS-QOL total score was plotted.

**Results:**

Mean changes from baseline to Week 26 for the IBS-QOL total and all subscale scores were significantly higher for patients treated with eluxadoline (both doses) compared to placebo. A significantly greater proportion of eluxadoline-treated patients were responders compared to placebo. Mean and mixed-effects model estimated mean IBS-QOL total scores were consistently higher for eluxadoline versus placebo over 52 weeks.

**Conclusions:**

Compared to placebo, twice-daily eluxadoline treatment significantly improved HRQOL among patients with IBS-D in two phase III trials.

## Introduction

Irritable bowel syndrome (IBS) is a chronic functional gastrointestinal disorder characterized by recurrent abdominal pain and altered bowel movements in the absence of structural, major inflammatory, or biochemical abnormalities [1, 2]. IBS subtypes are classified based on the predominant stool form: diarrhea predominant (IBS-D), constipation predominant (IBS-C), or mixed/alternating bowel patterns (IBS-M/IBS-A). IBS is a common disorder, with an estimated global prevalence of 11% in adults [3]; IBS-D accounts for approximately one-third of all IBS cases [4, 5].

The symptom burden experienced by patients with IBS-D, including diarrhea, abdominal pain, urgency, and bloating, has been shown to negatively impact patients’ health-related quality of life (HRQOL) and social functioning [6]. The burden associated with the symptoms of IBS-D not only results in lower HRQOL, but is also associated with impairments in everyday activities, missed work/school, and reduced productivity [7, 8]. Patients with IBS-D appear to have the lowest HRQOL compared to patients with other IBS subtypes [9], and impairments in HRQOL associated with IBS-D are comparable to or greater than those observed for other chronic diseases, such as asthma, gastroesophageal reflux disease, and migraine [8].

Management of IBS-D includes lifestyle/diet modifications, as well as use of both over-the-counter and prescription medications. Eluxadoline is a mixed µ-opioid receptor and κ-opioid receptor agonist and δ-opioid receptor antagonist approved by the US Food and Drug Administration and the European Medicines Agency for the treatment of IBS-D in adults [10–12]. In two phase III trials (IBS-3001 and IBS-3002), a significantly greater proportion of patients treated with eluxadoline 75 mg or 100 mg had simultaneous improvements in both abdominal pain and diarrhea compared to placebo [13]. Eluxadoline exhibited rapid onset of action, with efficacy observed as early as the first week of dosing and sustained over 6 months [14]. Eluxadoline was well tolerated; constipation and nausea were the most common adverse events [15]. On quality of life, eluxadoline was significantly superior to placebo after 12 weeks of treatment [13]. These data suggest that eluxadoline treatment has the potential to impact HRQOL in patients with IBS-D via improvement of the burdensome symptoms of IBS-D, including abdominal pain, diarrhea, and urgency [13, 16]. The objective of the current post hoc analyses was to further evaluate HRQOL in eluxadoline-treated patients with IBS-D based on pooled data from the two phase III trials.

## Methods

### Patient population and study design

HRQOL data were evaluated from two randomized, double-blind, placebo-controlled, parallel-group, multicenter studies (IBS-3001 [ClinicalTrial.gov identifier: NCT01553591] and IBS-3002 [NCT01553747]) for which clinical results have been reported previously [13–15]. In brief, adults aged 18–80 years meeting Rome III criteria [17] for IBS-D were eligible to participate if they reported an average worst abdominal pain score > 3.0 (on a scale of 0 [no pain] to 10 [worst imaginable pain]), an average Bristol Stool Form Scale score ≥ 5.5 (on a scale of 1 [hard stool] to 7 [watery diarrhea]), ≥ 5 days with a Bristol Stool Form Scale score ≥ 5, and an average IBS-D global symptom score ≥ 2.0 (on a scale of 0 [no symptoms] to 4 [very severe symptoms]) during the week prior to randomization.

In IBS-3001, eligible patients were randomized to receive an oral capsule of eluxadoline (75 mg or 100 mg) or placebo twice daily for 52 weeks; HRQOL assessments were captured during the entire 52-week treatment period. In IBS-3002, patients were randomized to receive the above treatments for 26 weeks followed by a 4-week, single-blind placebo withdrawal period (study completion at 30 weeks). HRQOL assessments were collected for the first 26 weeks and carried forward into the single-blind withdrawal period. HRQOL assessments completed during the withdrawal period in IBS-3002 were excluded from the current analyses. A detailed description of the study designs has been presented previously [13].

### Irritable Bowel Syndrome Quality of Life questionnaire

The Irritable Bowel Syndrome Quality of Life questionnaire (IBS-QOL) is a self-administered, disease-specific questionnaire designed to assess the burden of IBS on patients’ everyday functioning and well-being. It was developed based on a comprehensive review of the literature and with input from expert clinicians [18]. The instrument provides advantages over more general measures of HRQOL by capturing perceived quality of life specific to IBS. Numerous studies have documented the psychometric properties and confirmed internal consistency, reproducibility, validity, and responsiveness of the IBS-QOL [14, 17–20].

The IBS-QOL consists of 34 items assessing patients’ well-being across eight subscales: dysphoria (eight items); interference with activity (seven items); body image (four items); social reaction (four items); health worry (three items); food avoidance (three items); relationships (three items); and sexual (two items). Respondents rate each item on a 5-point response scale ranging from 1 (“Not at all”) to 5 (“Extremely”/“A great deal”), based on a 30-day recall period. All items are sum-scored to calculate the IBS-QOL total score, then transformed to a scale of 0–100 using the following formula: [(sum of items − lowest possible score)/possible raw score range] × 100 [18]. A higher score represents higher HRQOL.

Previous research has indicated a 10- to 14-point change in the IBS-QOL total score, which represents a clinically meaningful improvement in female patients with IBS, painful constipation, or chronic abdominal pain [21], while a more recent study suggested a higher threshold of a 17- to 20-point change could provide an improved definition of a responder when evaluating treatment effect on IBS-QOL score in an IBS-D patient population [19]. Accordingly, to evaluate a range of thresholds for responder definitions, thresholds of an improvement of ≥ 14 points and ≥ 20 points were pre-specified for exploration in the current analyses.

### IBS-QOL data collection and pooling

The IBS-QOL was completed by patients at baseline and Weeks 4, 8, 12, 18, and 26 in both trials, at Week 30 after the 4-week placebo withdrawal period in IBS-3002, and at Weeks 36, 44, and 52 in IBS-3001. Data were pooled from both IBS-3001 and IBS-3002 for all time points up to Week 26. As the Week 30 assessment in IBS-3002 was after a 4-week, single-blind placebo washout period, scores from this time point were not included.

### Statistical analyses

Descriptive statistics were used to assess patient characteristics and mean IBS-QOL total and subscale scores. Change from baseline to Week 26 was analyzed using an analysis of covariance model with treatment group and trial as factors and baseline score as covariate. The percentage of IBS-QOL responders (patients with a ≥ 14- or ≥ 20-point increase from baseline) was compared between treatment groups using chi-squared tests.

Mean IBS-QOL total scores over 52 weeks were also evaluated using a longitudinal mixed-effects model to minimize the potential for statistical bias when examining the observed raw values; the model was fitted to pooled data to estimate the treatment effects of eluxadoline. Longitudinal model terms included treatment group, time, treatment by time interaction, baseline score, and higher-order time effects. The higher-order time effects were included to account for the curvilinearity observed in the data. Empirical cumulative distribution functions (CDFs) for changes from baseline to Week 26 for IBS-QOL total score were plotted to further assess differences between eluxadoline and placebo groups. Missing data were not imputed; for longitudinal data, model fits employed direct likelihood methods, which under missingness at random assumptions, perform similarly to multiple imputations [22].

## Results

### Demographic and clinical characteristics

A total of 2423 patients were included in the pooled intent-to-treat population for analysis [13]. The mean age, sex, and race distributions were similar between the treatment groups; mean patient age ranged from 44.8 to 46.4 years, and the majority of patients were female (65.1–66.7%) and white (85.1–86.4%). Baseline disease characteristics were similar between all treatment groups, with an average abdominal pain score of 6.07–6.14 and an average of 4.78–4.95 bowel movements per day at baseline (Table 1).

**Table 1:** Demographics and baseline clinical characteristics for the pooled analysis cohort

Characteristic	Placebo (*n* = 809)	Eluxadoline 75 mg (*n* = 808)	Eluxadoline 100 mg (*n* = 806)
Mean age, years (SD)	46.4 (14.0)	44.8 (13.2)	45.0 (13.6)
Age ≥ 65 years, *n* (%)	102 (12.6)	65 (8.0)	74 (9.2)
Sex, *n* (%)			
Female	527 (65.1)	537 (66.5)	538 (66.7)
Male	282 (34.9)	271 (33.5)	268 (33.3)
Race, *n* (%)			
White	699 (86.4)	699 (86.5)	686 (85.1)
Black	89 (11.0)	92 (11.4)	96 (11.9)
Other	21 (2.6)	17 (2.1)	24 (3.0)
Baseline scores, mean (SD) [min, max]			
Abdominal pain^a^	6.14 (1.53) [3.1, 10.0]	6.07 (1.50) [3.1, 10.0]	6.07 (1.51) [3.1, 10.0]
Stool consistency^b^	6.24 (0.41) [5.4, 7.0]	6.25 (0.40) [5.5, 7.0]	6.25 (0.42) [5.5, 7.0]
Number of bowel movements per day	4.85 (2.52) [0.9, 33.6]	4.78 (2.53) [1.0, 29.4]	4.95 (3.60) [1.0, 75.0]
IBS-QOL^c^	45.3 (23.1) [0, 100]	48.3 (23.3) [0, 100]	47.2 (23.0) [0, 100]

*IBS-QOL* Irritable Bowel Syndrome Quality of Life questionnaire, *SD* standard deviation

^a^Scale of 0–10

^b^Scale of 1–7

^c^Scale of 0–100

### Change from baseline in IBS-QOL total and subscale scores

**Fig. 1:**
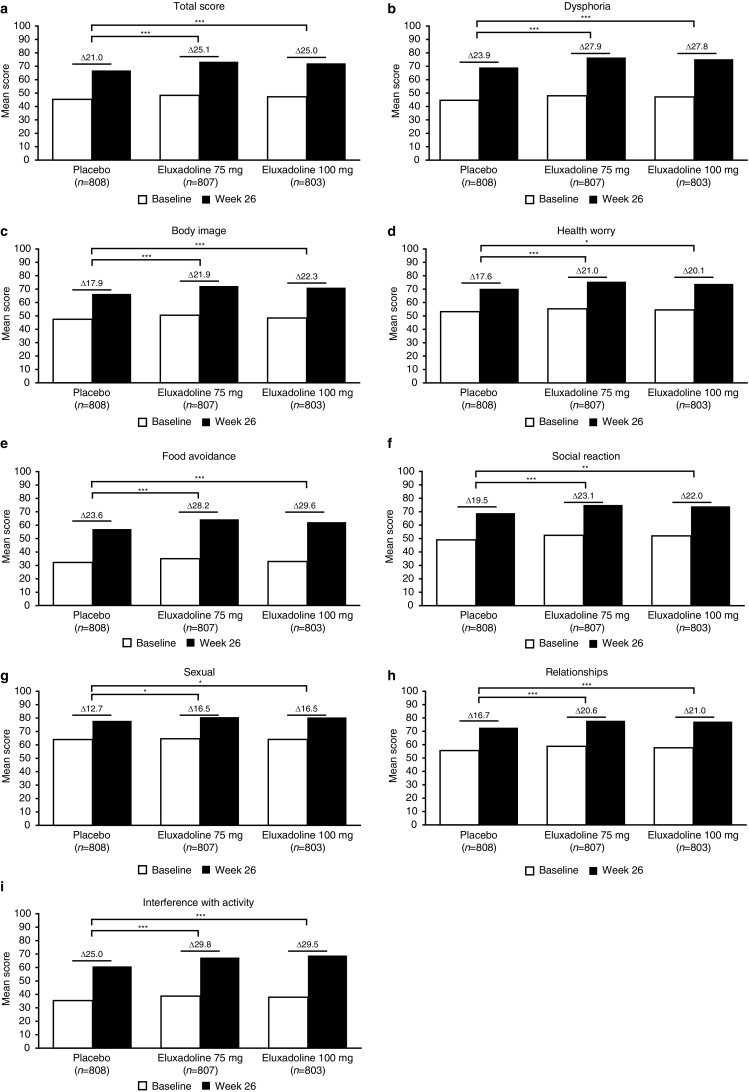
Mean IBS-QOL total and subscale scores at baseline and Week 26 for the eluxadoline 75 mg and 100 mg treatment groups versus placebo. Mean differences were calculated for each treatment group, and comparisons were made between the different treatment groups. *IBS-QOL* Irritable Bowel Syndrome Quality of Life questionnaire. *p ≤ 0.05, **p < 0.01, ***p < 0.001 versus placebo, analysis of covariance

Mean change in IBS-QOL total score from baseline to Week 26 was significantly higher for patients treated with eluxadoline 75 mg (25.1) and 100 mg (25.0) compared to those treated with placebo (21.0, both *p* < 0.001) (Fig. 1a). Patients treated with eluxadoline 75 mg and 100 mg also had significantly greater increases from baseline to Week 26 for all eight subscale scores: dysphoria, body image, food avoidance, relationships, interference with activity (all *p* < 0.001), health worry (*p* < 0.001 and *p* ≤ 0.05, respectively), social reaction (*p* < 0.001 and *p* < 0.01, respectively), and sexual (both *p* ≤ 0.05) (Fig. 1b–i).

A significantly greater proportion of eluxadoline-treated patients were IBS-QOL responders compared to placebo for both responder definitions evaluated. At a threshold of ≥ 14-point change from baseline, 67.1% (*p* < 0.01) and 64.6% (*p* < 0.05) of patients treated with eluxadoline 75 mg and 100 mg, respectively, were responders, compared to 58.0% in the placebo group. Similarly, at a threshold of ≥ 20-point change from baseline, 55.8% (*p* < 0.01) and 56.8% (*p* < 0.01) of patients treated with eluxadoline 75 mg and 100 mg, respectively, were responders, compared to 47.5% for placebo (Table 2).

**Table 2:** IBS-QOL responder analyses

Proportion of patients, %	≥ 14-point change Eluxadoline			≥ 20-point change Eluxadoline		
	Placebo (*n* = 809)	75 mg (*n* = 808)	100 mg (*n* = 806)	Placebo (*n* = 809)	75 mg (*n* = 808)	100 mg (*n* = 806)
IBS-QOL total score	58.0	67.1**	64.6*	47.5	55.8**	56.8**
Dysphoria	60.4	65.4	65.7	51.2	58.1*	56.8
Body image	48.8	57.6**	55.5*	39.9	48.4**	46.3*
Health worry	56.4	62.7*	58.5	42.4	47.2	45.2
Food avoidance	61.0	66.7*	67.3*	50.0	55.8	59.3**
Social reaction	49.8	57.2*	56.9*	42.2	48.1	48.2*
Sexual	34.6	42.0*	40.2	34.6	42.0*	40.2
Relationships	53.9	60.0*	59.4	39.4	45.2	46.4*
Interference with activity	65.9	72.2*	70.1	54.8	62.0*	61.7*

Bolded values indicate significance

*IBS-QOL* Irritable Bowel Syndrome Quality of Life questionnaire

*p < 0.05, **p < 0.01 versus placebo, Chi-squared test

For subscale scores at the ≥ 14-point change, significantly more eluxadoline-treated patients were responders compared to placebo on seven of eight subscales (all except dysphoria) and three of eight subscales (body image, food avoidance, and social reaction) for the eluxadoline 75 mg and 100 mg groups, respectively (Table 2). Similar results were observed for the higher ≥ 20-point change from baseline responder definition, with a significantly higher proportion of responders observed in four of eight subscales (dysphoria, body image, sexual, and interference with activity) and five of eight subscales (body image, food avoidance, social reaction, relationships, and interference with activity) for the eluxadoline 75 mg and 100 mg groups, respectively.

### IBS-QOL total score model estimates and change over time

**Fig. 2:**
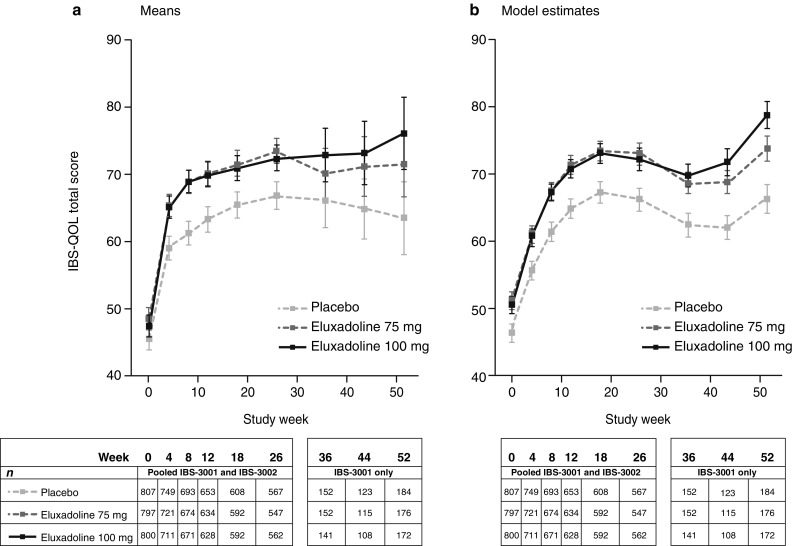
Longitudinal data on **a** mean IBS-QOL total scores and **b** mixed-effects model estimated means with 95% confidence intervals for the eluxadoline 75 mg and 100 mg treatment groups versus placebo. Data pooled from IBS-3001 and IBS-3002 for all time points up to Week 26; Weeks 36–52, IBS-3001 only. *IBS-QOL* Irritable Bowel Syndrome Quality of Life questionnaire

Longitudinal data on mean IBS-QOL total scores and mixed-effects model estimated means showed patients treated with eluxadoline 75 mg and 100 mg had consistently higher IBS-QOL total scores over 52 weeks of treatment compared to placebo (Fig. 2). IBS-QOL total score means continued to increase over 52 weeks of treatment in patients treated with eluxadoline, while the mean IBS-QOL total score for patients treated with placebo decreased from Weeks 26 to 52 (Fig. 2a). The model estimates of the IBS-QOL total score for all groups increased up to Week 52, with a plateau observed between Weeks 18 and 44 (Fig. 2b).

**Fig. 3:**
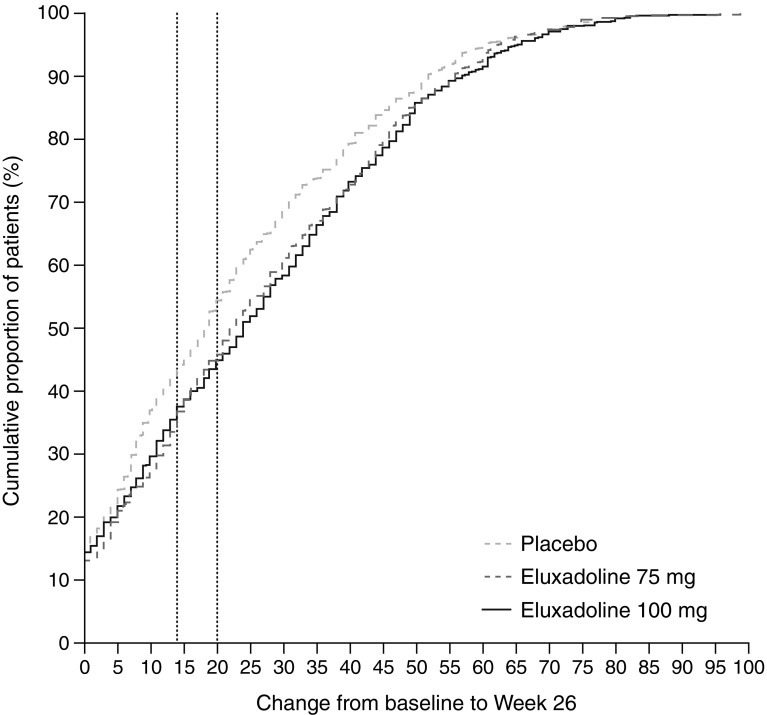
Cumulative distribution function plots of changes from baseline to Week 26 for IBS-QOL total scores for the eluxadoline 75 mg and 100 mg treatment groups versus placebo. *IBS-QOL* Irritable Bowel Syndrome Quality of Life questionnaire

A CDF plot of change from baseline scores at Week 26 showed that more patients treated with eluxadoline 75 mg and 100 mg had higher increases in IBS-QOL total score compared to patients who received placebo (Fig. 3). Consistent separation was observed between the placebo and both eluxadoline groups, initially visible from the 5-point change threshold and beyond, with clear delineation at both the 14- and 20-point change thresholds.

## Discussion

Prior studies have highlighted significant physical and emotional impairments in HRQOL experienced by patients with IBS [8, 9, 23]. Reductions in quality of life may be primarily due to the symptom burden experienced by these patients, including abdominal pain, bloating, diarrhea, and urgency related to bowel movements [6, 23]. Previous research comparing HRQOL between patients with different chronic diseases found patients with IBS had significantly lower scores on the Medical Outcomes Study 36-item Short-Form Health Survey, indicating lower HRQOL compared to other chronic disorders, including gastroesophageal reflux disease, dyspepsia, asthma, and migraine [24].

In these two phase III clinical trials of eluxadoline (IBS-3001 and IBS-3002), HRQOL was assessed among patients with IBS-D using the IBS-QOL, a disease-specific HRQOL instrument which aims to provide a comprehensive view of the disease from emotional, social, and physical perspectives [13, 24]. As previously reported [13], patients receiving eluxadoline 75 mg and 100 mg had significantly greater changes from baseline in their IBS-QOL total score at Week 12 compared with placebo (22.1 and 22.8 vs. 17.8, respectively), suggesting eluxadoline treatment is associated with improved HRQOL [13]. The current analyses present a more in-depth analysis of HRQOL data collected from the two phase III eluxadoline trials beyond mean IBS-QOL total score and change of mean IBS-QOL total score from baseline at Week 12; an understanding of change at the individual level (e.g., responder analyses) versus group-level changes and long-term effects of eluxadoline treatment on HRQOL are critical.

Significantly greater improvements from baseline to Week 26 were observed for eluxadoline-treated patients compared to placebo for the IBS-QOL total score and all eight subscale scores, thus indicating that eluxadoline had a significant and positive impact on patients’ HRQOL over the treatment period. This may be due to the efficacy of eluxadoline in improving symptoms [13]. Improvements in symptoms such as abdominal pain and stool consistency may decrease the impact of IBS-D on patients in several elements assessed in the IBS-QOL such as health worry, food avoidance, and interference with activity, as well as impact on interpersonal relations (e.g., social reaction, relationships, and sexual). Symptom improvement may also relieve the emotional impact of IBS-D, which may lead to improvements in body image and dysphoria. IBS-QOL scores have been shown to be significantly correlated with psychological and pharmacological treatment effects and can be used to differentiate between treatment responders and non-responders [21].

A 10- to 14-point change in IBS-QOL score is generally considered to be clinically meaningful based on a heterogeneous set of patients with a variety of functional bowel disorders [21]. A study of approximately 750 patients with IBS-D suggested a criterion of 17- to 20-point change may improve the definition of a responder in clinical trials for IBS-D treatments, although no assessment was made regarding whether this criterion indicated a clinically meaningful change [19]. In the current analyses, a significantly greater proportion of eluxadoline-treated patients achieved IBS-QOL responder status at the traditional 14-point threshold previously defined as clinically meaningful [21] and at the more conservative 20-point threshold [19]. CDF analyses supported the total score findings, further substantiating the observed effects of eluxadoline in the primary clinical trial analyses as clinically meaningful.

The longitudinal analysis demonstrated statistically significant improvements in IBS-QOL total score for eluxadoline versus placebo, with improvements observed at 4 weeks and maintained over 52 weeks of treatment. These results are consistent with other endpoints in the clinical trials, where eluxadoline significantly improved IBS-D symptoms with sustained efficacy observed over 6 months [13, 14]. Overall improvements observed in HRQOL in these analyses, as measured by the IBS-QOL, are consistent with the efficacy of eluxadoline in alleviating IBS-D symptoms.

### Limitations

Although the 30-day patient recall period used in the IBS-QOL is considered acceptable for health outcomes assessments and has been tested in prior IBS-QOL validation studies [21], a shorter recall period may improve the accuracy of patient responses. Furthermore, these analyses were conducted among patients meeting Rome III criteria for IBS-D; therefore, results may not be generalizable to all patients with IBS-D in the general population. Additionally, due to differences in trial design between IBS-3001 and IBS-3002, pooled data were only available for up to 26 weeks; longer-term impacts of treatment with eluxadoline over 52 weeks could only be evaluated based on data from a single trial, in which decreased sample size may result in potential bias.

## Conclusions

IBS-D continues to represent a significant burden for patients, highlighting a need for appropriate therapies that effectively manage the multiple chronic symptoms of IBS-D and improve patients’ HRQOL. In phase III trials, twice-daily treatment with eluxadoline was associated with significant improvements in HRQOL compared to placebo among adult patients with IBS-D, with improvements observed at 4 weeks and maintained over 52 weeks of treatment.
